# Endemicity of Yaws and Seroprevalence of *Treponema pallidum* Antibodies in Nonhuman Primates, Kenya

**DOI:** 10.3201/eid2511.190716

**Published:** 2019-11

**Authors:** Dawn M. Zimmerman, Emily H. Hardgrove, Michael E. von Fricken, Joseph Kamau, Daniel Chai, Samson Mutura, Velma Kivali, Fatima Hussein, Peris Ambala, Andrea Surmat, Joseph G. Maina, Sascha Knauf

**Affiliations:** Smithsonian Conservation Biology Institute, Washington DC, USA (D.M. Zimmerman, E.H. Hardgrove, M.E. von Fricken);; George Mason University, Fairfax, Virginia, USA (D.M. Zimmerman, M.E. von Fricken);; Virginia Polytechnic Institute and State University, Blacksburg, Virginia, USA (E.H. Hardgrove);; Institute of Primate Research, Nairobi, Kenya (J. Kamau, D. Chai, S. Mutura, F. Hussein, P. Ambala);; International Livestock Research Institute, Nairobi (V. Kivali);; Mpala Research Centre and Wildlife Foundation, Nanyuki, Kenya (A. Surmat);; Kenya Wildlife Service, Nairobi (J.G. Maina);; German Primate Center, Goettingen, Germany (S. Knauf)

**Keywords:** Treponema pallidum, bacteria, yaws, antibodies, baboon, Kenya, primate, vervet monkey, nonhuman primates, zoonoses, Laikipia County, Kenya

## Abstract

Human yaws has historically been endemic to Kenya, but current epidemiologic data are lacking. We report seroprevalence for *Treponema pallidum* antibodies in olive baboons (*Papio anubis*) and vervet monkeys (*Chlorocebus pygerythrus*) in Laikipia County, Kenya. Our results suggest endemicity of the yaws bacterium in monkeys, posing a possible zoonotic threat to humans.

Yaws is a disease caused by the bacterium *Treponema pallidum* subsp. *pertenue*, which is believed to be an exclusively human pathogen (*1*). However, this bacterium has recently been identified in African nonhuman primates (NHPs) (*2*), raising concerns about a possible zoonotic reservoir for human infection. Kenya is 1 of 76 countries that the World Health Organization categorizes as previously endemic for yaws, but no current data support its presence or absence (http://apps.who.int/gho/data/node.main.NTDYAWSEND). However, sustainable yaws eradication will rely on information about transmission dynamics and potential links between human and NHP *T. pallidum* strains (*3*).

In the early 1960s, Fribourg-Blanc and Mollaret tested 150 serum samples from wild-caught baboons (*Papio* sp.) from Guinea and Kenya (*4*). Although 72 (65%) of 111 serum samples from Guinea were positive for *T. pallidum* antibodies, none of the samples from Kenya were positive. In subsequent years, an additional 276 serum samples from baboons in Kenya supported the absence of *T. pallidum* infection. However, a more recent study of baboon samples collected during 1977–1994 in Kenya reported serologic evidence of *T. pallidum* infection in Nanyuki, Laikipia County (prevalence 57.5%) (*5*). For our study, we hypothesized that 39 years after the first samples were positive for antibodies against *T. pallidum* in Nanyuki (*5*), infection is still present in the NHP population.

All animal protocols were approved by the Kenya Wildlife Service (permit #4004), the Institute of Primate Research Scientific and Ethics Review Committee, and the Smithsonian Institution Animal Use and Care Committee. In October 2016, we sampled 65 olive baboons (*Papio anubis*) and 2 vervet monkeys (*Chlorocebus pygerythrus*) at sites surrounding the Mpala Research Centre in Laikipia County, Kenya. We performed a preliminary serologic screening by using the immunochromatographic Dual Path Platform (DPP) HIV-Syphilis Assay (Chembio Diagnostic Systems, Inc., http://chembio.com) according to the manufacturer guidelines. This syphilis (*T. pallidum*) assay is a useful screening tool because antibodies against *Treponema* subspecies are cross-reactive (*6*). We tested 67 samples with the DPP assay; 49 were positive and 18 negative.

However, because this test is not certified for use with NHPs, we subsequently confirmed results by using the *T. pallidum* Particle Agglutination Assay (TPPA) (SERODIA TPPA, https://www.fujirebio-us.com), which has been validated for use in baboons (*7*). Of the 52 samples tested with the TPPA assay, there were 33 positive, 6 negative, and 13 inconclusive results. Inconclusive TPPA results indicate nonspecific antibodies reacting with nonsensitized particles. Because of limited sample material, we were unable to perform repeated testing with a preabsorption step to remove all nonspecific binding antibodies (as described in the assay manual) and therefore excluded the inconclusive TPPA results from our analysis.

If we defined seropositive monkeys as those with positive results for the TPPA or DPP, 1 of 2 vervet monkeys and 53 (85.5%) of 62 baboons were seropositive. Male baboons (90.4%, 38/42) had a relative seropositivity risk ratio of 1.3 (95% CI 0.984–1.858) when compared with female baboons (72.2%, 13/18); however, this difference was not significant (p = 0.111 by Fisher exact test). If we included age, in addition to sex, in the analysis, adult male and female baboons both showed 100% seropositivity (21/21 and 10/10, respectively). Subadult males and females also showed seropositivity of 100% (6/6 and 1/1, respectively). Juveniles had a combined seropositivity of 61.1%: a total of 81.8% (9/11) of males and 28.6% (2/7) of females were seropositive. Infants had the lowest seroprevalence rate (50%, 2/4) (Table).

**Table:** Demographic data and serologic results for nonhuman primates sampled for *Treponema pallidum* antibodies, Laikipia County, Kenya, October 2016*

Species, age group†	No. positive/no. tested (%)	
	Male	Female
Olive baboon (*Papio anubis*)		
Adult	21/21 (100)	10/10 (100)
Subadult	6/6 (100)	1/1 (100)
Juvenile	9/11 (82)	2/7 (29)
Infant	2/4 (50)	ND
Subtotal	38/42 (90)	13/18 (72)
Vervet monkey (*Chlorocebus pygerythrus*)		
Adult	0/1 (0)	ND
Juvenile	1/1 (100)	ND
Subtotal	1/2 (50)	ND
Total	39/44 (89)	13/18 (72)

*Samples were tested by using the Dual Path Platform Assay or the *Treponema pallidum* Particle Agglutination Assay. ND, not done. †Age ranges for *P. anubis* baboons, infant, <1.3 y; male juvenile, 1.3–6 y; female juvenile, 1.3–5 y; male subadult, 6–9 y; female subadult 5–6 y; male adult, >10 y; female adult, >6 y (Appendix reference *1*). Age ranges for *C. pygerythrus* monkeys: juvenile, 22–40 mo; adult, >40 months (Appendix reference *2*).

None of the tested NHPs had overt clinical signs of infection, such as skin lesions, which might have contained *T. pallidum* DNA. However, several other studies found that NHPs are frequently seropositive for *T. pallidum* antibodies without clinical lesions (*5**,**8**,**9*). Because wild NHPs are not treated and bacterial clearance is unlikely, the absence of lesions presumably corresponds to the latency stage of infection, which is also a key characteristic of human treponematoses (*10*). Future molecular investigations should include nontreponemal tests to further support the assumption that animals are in the latency stage and should target the DNA of the pathogen, which would enable comparison of *T. pallidum* strains of NHP origin from Kenya with those infecting NHPs in neighboring countries and possibly humans. In Tanzania, a country that has a similar history of previous yaws endemicity in humans and lacks current prevalence data, clinical lesions have been documented in olive baboons, vervet monkeys, yellow baboons, and blue monkeys, in addition to widespread seroprevalence in NHPs closely matching previous human infection geographic distribution (*9*).

Our results suggest that evidence of *Treponema* exposure in NHPs continues to be present in Laikipia County almost 4 decades after it was first detected. Our data provide further evidence that, in East Africa, *T. pallidum* infection is endemic to NHPs and that multiple NHP taxa contain antibodies indicating latent infection. Providing reliable information on the epidemiology of treponematoses in humans and NHPs has major programmatic implications for yaws eradication. Under a One Health approach, we call for additional yaws surveillance in communities in Kenya, especially in regions where NHPs and humans coexist.

Appendix Additional information (2 references) for endemicity of yaws shown by *Treponema pallidum* antibodies in nonhuman primates, Kenya.
