# Supplementary material for: Endemicity of Yaws and Seroprevalence of Treponema pallidum Antibodies in Nonhuman Primates, Kenya
Source: Emerg Infect Dis. 2019 Nov;25(11):2147–9. doi: 10.3201/eid2511.190716 (PMC6810213; doi:10.3201/eid2511.190716)
Supplement: Appendix — Additional information (2 references) for endemicity of yaws shown by Treponema pallidum antibodies in nonhuman primates, Kenya. [file 19-0716-Techapp-s1.pdf]

Article DOI: https://doi.org/10.3201/eid2511.190716

Endemicity of Yaws and *Treponema pallidum* Antibodies in Nonhuman Primates, Kenya

Appendix

References

1. Altmann J, Altmann S, Hausfater G. Physical maturation and age estimates of yellow baboons, *Papio cynocephalus*, in Amboseli National Park, Kenya. Am J Primatol. 1981;1:389–99. https://doi.org/10.1002/ajp.1350010404

2. Turner TR, Schmitt CA, Cramer JD, Lorenz J, Grobler JP, Jolly CJ, et al. Morphological variation in the genus *Chlorocebus*: ecogeographic and anthropogenically mediated variation in body mass, postcranial morphology, and growth. Am J Phys Anthropol. 2018;166:682–707. PubMed https://doi.org/10.1002/ajpa.23459
